# Modality use in joint attention between hearing parents and deaf children

**DOI:** 10.3389/fpsyg.2015.01556

**Published:** 2015-10-12

**Authors:** Nicole Depowski, Homer Abaya, John Oghalai, Heather Bortfeld

**Affiliations:** ^1^Department of Psychology, University of Connecticut, StorrsCT, USA; ^2^Head and Neck Surgery, Department of Otolaryngology, Stanford University School of Medicine, StanfordCA, USA; ^3^Psychological Sciences, University of California, MercedMerced, CA, USA

**Keywords:** joint attention, multimodal communication, Parent-child communication, ELAN, cochlear implants, deaf

## Abstract

The present study examined differences in modality use during episodes of joint attention between hearing parent-hearing child dyads and hearing parent-deaf child dyads. Hearing children were age-matched to deaf children. Dyads were video recorded in a free play session with analyses focused on uni- and multimodality use during joint attention episodes. Results revealed that adults in hearing parent-deaf child dyads spent a significantly greater proportion of time interacting with their children using multiple communicative modalities than adults in hearing parent-hearing child dyads, who tended to use the auditory modality (e.g., oral language) most often. While these findings demonstrate that hearing parents accommodate their children’s hearing status, we observed greater overall time spent in joint attention in hearing parent-hearing child dyads than hearing parent-deaf child dyads. Our results point to important avenues for future research on how parents can better accommodate their child’s hearing status through the use of multimodal communication strategies.

## Introduction

Imagine a world in which the way that people communicate is inherently different from how you communicate: You use visual information and they insist on using auditory information. The result is confusion and miscommunication. For many children who are born deaf, this is the reality they initially face. This is because 90 percent of deaf children are born to hearing parents (Mitchell and Karchmer, 2004), meaning that there is an inherent mismatch between parent and child in the dominant modality used for communication. Here we examine how hearing parents accommodate their deaf children’s hearing status by documenting the modality or modalities used in communication between parents and children. To what extent do parents use changes in modality to accommodate their child’s hearing loss and how do children adapt to those changes?

Language development is often delayed in deaf children of hearing parents (Lederberg and Everhart, 1998) because the majority of hearing parents of deaf children have no prior experience using sign language to communicate (DeMarco et al., 2007) and must adjust to their child’s hearing status. Parents may choose to learn sign language, they may choose to have their deaf child evaluated for cochlear implant candidacy, or they may do both of these things. Regardless, early communication between hearing parents and deaf children presents a significant obstacle, as suggested by evidence that deaf children of hearing parents have an increased rate of behavioral issues and that these issues are related to communication difficulties (Barker et al., 2009).

This mismatch may pose difficulties for parents as well. Hearing parents of deaf children may experience stress specifically with regard to their child’s deafness (Lederberg and Golbach, 2002), and those with the highest levels of stress also tend to have deaf children with more social and emotional development problems (Hitermair, 2006). Given the clear impact of maternal stress on children’s development and the importance of communication between parents and their children to mitigate sources of stress, the present study was designed to compare communication in hearing parent-deaf child dyads (in which parents were using a predominantly auditory-oral approach) and hearing parent-hearing child dyads. One way that hearing mothers appear to mitigate the difficulties in communication with their deaf children is by changing their own behavior to accommodate the children’s limited access to the auditory modality. For example, during free play sessions, hearing mothers of deaf infants have been shown to use exaggerated gestures relative to deaf mothers of deaf children (Koester et al., 1998a), suggesting that they are trying to use a non-auditory modality to communicate even if they are not learning sign themselves. In another study, hearing mothers of deaf infants were found to move objects into a child’s visual field and tap on or point to objects to get the child to attend to them (Waxman and Spencer, 1997). Our goal in the current study was to characterize how hearing parents of deaf children who use an auditory-oral approach accommodate their children’s communicative needs.

Research that is informative on the issue of communicative accommodation involves use of the Still Face Paradigm, in which a mother is instructed to maintain a neutral, unemotive face at prescribed intervals during normal interaction with her infant (Cohn and Tronick, 1983). Although this paradigm was initially developed for the study of effects of depressive mothers on young children (Cohn and Tronick, 1983), it has since been used to probe other areas of early development (see Mesman et al., 2009). Typically, when the mother tries to re-engage with the infant after a period of maintaining a blank face, she must work harder than usual to successfully re-engage with her infant. When used with deaf children, the paradigm has revealed that hearing mothers use spoken language to engage their 9-month-old infants more than deaf mother-deaf child dyads (Koester et al., 1998b), despite the child’s lack of access to the auditory modality. While this is not entirely surprising, no difference between dyad types was found in use of the visual or tactile modalities to re-engage the infants (Koester et al., 1998b; Koester, 2001), showing that hearing parents were accommodating their deaf infants communicatively.

Another way to examine whether and how parents accommodate their children communicatively is through their efforts to establish joint attention. Joint attention is the ability to focus simultaneously on an object or event and another person, sometimes described as “shared intentionality” (Tomasello, 1995; Tomasello and Carpenter, 2007). Joint attention can be further divided into the acts of both initiating a bid for attention (e.g., pointing at a balloon) and responding to a bid for attention (e.g., commenting that the balloon is red; Mundy et al., 2007). This is an act of reciprocating communication within a dyad, and is essential to basic human communication. The act of successfully initiating joint attention is more sophisticated than responding to it; thus, the act of a child successfully initiating joint attention can be considered to be the start of formalized and intentional communication (Brinck, 2001) Although joint attention is commonly the focus of research on language development, it is also relevant to more general social and emotional development (Mundy et al., 1990; Corkum and Moore, 1998; Mundy and Gomes, 1998; Mundy and Neal, 2000). Given these broad developmental implications, joint attention provides a way to characterize interactions between hearing parents and their deaf children.

Gale and Schick (2009) focused on symbol-infused joint attention or joint attention during symbolic communication, between 24-month-old deaf children and their hearing parents. Although deaf children of hearing parents did not differ from deaf children of deaf parents or hearing children of hearing parents on most language measures, they did engage in significantly fewer sustained interactions (Gale and Schick, 2009). This difference is notable given that much of the cognitive benefit derived from joint attention originates from the sustained interaction between parent and child, suggesting that this may be the source of some of the negative developmental outcomes seem in this population. In other research, hearing parents of hearing children (between 18 and 36 months of age) rated their children as having higher adaptive social behavior than hearing parents of deaf children of the same age range. Moreover, these researchers found that higher rates of successful joint attention were associated with higher ratings of the children’s adaptive social behavior, regardless of hearing status (Nowakowski et al., 2009). This highlights the substantial role that joint attention plays in development in general. Importantly, hearing mothers of deaf 36-month-olds have been shown to use more modalities of communication to gain their child’s attention during interaction than hearing parents of hearing children do (Lederberg and Everhart, 1998).

While the comparison of hearing parent-deaf child dyads to hearing parent-hearing child dyads is helpful, it is just as important to compare modality-matched dyads (e.g., hearing parent-hearing child dyads and deaf parent-deaf child dyads). Lieberman et al. (2014) did just this, focusing on the specific types of gaze used by these dyads during joint attention. Their results demonstrate that the way in which partners in these different dyads engage one another is qualitatively different. Deaf children switched gaze between the parent and the object of interest much more often than hearing parents of hearing children, suggesting that deaf children who are exposed to sign [in this case, American Sign Language (ASL)] are able meet the attention-switching requirements of joint attention (Lieberman et al., 2014). Compared to hearing parent-hearing child dyads, hearing parent-deaf child dyads spent less time overall in joint attention (Prezbindowski et al., 1998). Considering that much of the benefit of joint attention derives from the interaction inherent in it, and much of what is learned in joint attention can be symbolic (including language), this difference is of concern. The authors hypothesized that the reason for this is that hearing parents of deaf children try to engage their children in symbol-infused joint attention by using oral language (i.e., the auditory modality; Prezbindowski et al., 1998).

A hearing parent’s use of the auditory modality with a deaf child highlights one of the primary difficulties in hearing parent-deaf child communication. Hearing parents rely on oral language in the rest of their lives but cannot use it to communicate with their children effectively. While this may seem obvious, the instinctive use of oral communication by hearing parents affects important basic interactions, such as when parents direct their children’s attention to objects and events in their surroundings. In a study on children’s visual perception of Manual Coded English (MCE), a communication method which involves a hearing mother speaking while signing to her deaf child, mothers who used more deaf-friendly means of communication had children who saw more complete versions of the mothers’ signed utterances (Swisher, 1991). For example, the most successful mother, as measured by the percentage of complete utterances seen by her child, tapped the child to ensure that the child was paying attention to before the mother began to sign and did so more frequently than any other mother in the study (Swisher, 1991). This study is relevant to the current study as it highlights the link between language and joint attention. For deaf children, attention established in the visual modality is necessary for subsequent access to visual language. Even if a hearing parent makes the decision to have a deaf child implanted, there will be a period of time—that is, the preimplantation period—during which the dominant modality of communication is mismatched between parents and child.

Clearly, which modalities are used in communication between hearing parents and deaf children is a topic that merits further research. While previous research (e.g., Trautman, 2009) has examined modality differences in communication between hearing parents and deaf children in broad terms, in the present study we sought to establish more precise coding of modality use during establishment of joint attention between hearing parent and deaf children and compare that to similarly precise coding of hearing parent-hearing child dyads. We were particularly interested in seeing whether differences emerged between the two dyad types in terms of how the parent worked to establish a child’s attention. More generally, this study represents a first step toward documentation of the communicative modalities that non-signing hearing parents use to establish joint attention with their deaf children.

## Materials and Methods

### Participants

Four severely to profoundly deaf children (*n* = 4 females) aged 18.2–36.7 months (*M* = 26.83, *SD* = 7.78; specifically, ages 18.2, 24.1, 28.3, 36.7) and their hearing parents (*n* = 4 females), participated in the study. While all children were candidates for cochlear implantation, none of them had received an implant; the children were being instructed predominantly using the oral method. None of the children produced any spoken or signed language during videotaping in our sample. Each child was receiving at least 1 h per week of speech therapy, as well as some basic instruction in ASL. In addition, four hearing children (*n* = 4 females) ages 18.3–36.7 months (*M* = 26.85, *SD* = 7.72; specifically, 18.3, 24.1, 28.3, 36.7) and their hearing parents (*n* = 4 females) took part in the study. Participants were aged-matched, and were from the Southwestern and Northeastern United States. Each was recruited via the National Institute of Health website or local recruitment. The sample was primarily Caucasian (two of the deaf children identified as Caucasian, Hispanic/Latino), and all but one parent had completed at least high school. This study was carried out in accordance with the recommendations of the University of Connecticut Institutional Review Board and the Stanford University School of Medicine Institutional Review Board with written informed consent from all subjects. For participants who were young children, parents provided written informed consent. All subjects gave written informed consent in accordance with the Declaration of Helsinki.

### Materials

Age appropriate toys (a ball, a set of large blocks, a set of stacking cups, tableware, a tower of stacking rings, and toy cars) were used during a free-play session between the child and his/her primary caregiver, which occurred as part of a visit with a speech language pathologist (deaf children) or to the Husky Pup Language Lab at the University of Connecticut (hearing children). The speech language pathologist or experimenter instructed the caregiver to play with the child as she would at home; play sessions were video recorded for approximately five minutes (*M* = 464.23, *SD* = 154.35; see **Table 1**). Videos of hearing parent-deaf child dyads were then transmitted from collaborators at Stanford University to researchers at University of Connecticut using Research Electronic Data Capture (REDCap) electronic data capture tools hosted at Stanford University (Harris et al., 2009). REDCap is a secure, web-based application designed to support data capture for research studies. It provided the two labs with a vehicle for validated data entry with audit trails for tracking data entry and export, as well as procedures for importing data from external sources. For the current study, REDCap was used solely as a means of secure transfer of videos between collaborators, and was not used for any analytical/coding purposes.

**Table 1:** Lengths of play sessions (in seconds), proportion of time spent in joint attention episodes, and length of joint attention episodes (in seconds).

Dyad/age (in months)	Dyad 1 (18 m)	Dyad 2 (24 m)	Dyad 3 (28 m)	Dyad 4 (36 m)
Hearing parent-hearing child length of play session	385.44	540.76	679.84	408.58
*Total time (in seconds)*				
Hearing parent-deaf child length of play session	375.98	247.77	427.15	655.97
*Total time (in seconds)*				
Hearing parent-hearing child joint attention	0.12/48.02	0.34/184.76	0.19/129.34	0.55/225.28
*Proportion/length of time (in seconds)*				
Hearing parent-deaf child joint attention	0.11/39.79	0.19/43.25	0.38/158.29	0.02/12.34
*Proportion/length of time (in seconds)*				

### Procedure

The videos were coded for joint attention using ELAN (Wittenburg et al., 2006), language annotation software created at the Max Planck Institute for Psycholinguistics, (The Language Archive, Nijmegen, The Netherlands). ELAN allows for multimodal analyses of language and other behavior (http://tla.mpi.nl/tools/tla-tools/elan/), and is available free of charge. We use coding criteria for joint attention based on the work of Tek (2010), which was a modified version of the Early Social Communication Scales, a measure of early development that can be used on typically developing populations (Mundy et al., 1996.) Coded variables were analyzed using ELAN, Microsoft Excel, VassarStats, and SPSS.

#### Video Processing

Videos were reviewed for visual clarity and Adobe Premiere Pro (CS6) was used to cut the video to the start and end time of the play session. The start time of the play session was at the first frame in which the testing room’s door was closed, leaving the child and parent alone. The end of the play session was at the first frame in which the experimenter opened the door to end the play session. These two values were subtracted to give a baseline length of time for the play session. Next, intervals in which the video was uncodeable were marked. An uncodeable interval was defined as an interval of at least 5 s in which at least one participant’s face was not visible. The amount of uncodeable time was subtracted from the baseline length of time to yield a total length of play session for each participant.

#### Joint Attention Coding

Very few instances of child-initiated joint attention were observed; thus, this construct was not included for analysis in the paper. Moreover, in the present study, only successful bids for joint attention (i.e., joint attention episodes) were coded and quantified. A successful joint attention episode involved the adult making a bid for the child’s attention using pointing, gaze switching between the object and the child, tapping or touching the child, deliberate waving in the child’s visual field, changing affect, and/or language; this bid was then responded to by the child using pointing, gaze switching between the object and the parent, tapping or touching the parent, grasping the object of interest, deliberate waving in the parent’s visual field, changing affect, and/or language. This type of episode could also occur if a parent shifted the child’s attention from one object to another using the previously mentioned techniques. Any indication of the auditory modality being used is *during* its use within a joint attention episode. Most instances of use of the auditory modality with deaf children were brief and the hearing parent would proceed to a different modality of engagement.

To record joint attention in ELAN, a 5 s “rule of engagement” was followed (i.e., after interacting with an object, a member of the dyad had 5 s to begin to engage with the other member of the dyad and vice versa for interactions beginning with a member of the dyad). Similarly, there was a 5 s rule of disengagement, i.e., a joint attention episode was deemed to be terminated after neither participant engaged in joint attention behavior for 5 s. If either participant re-engaged within the 5-s window, the length of the episode was extended; the episode ended at the start of the first period of 5 s that displayed no joint attention behaviors.

#### Coding for Modality

All successful, adult-initiated joint attention episodes were then coded separately for both the parent’s and the child’s uses of the following modalities: auditory, visual, tactile, auditory-visual, auditory-tactile, visual-tactile, and auditory-visual tactile. The criteria are as follows. One episode could have multiple modalities used within it, as specified by the following categories:

##### Auditory

Behaviors in the auditory modality involved using sound to gain the attention of the other member of the dyad. These included language, humming, other vocal sounds (e.g., “psst!”), hitting an object to make noise, clapping (if the other member of the dyad was unable to see the clap), and causing a toy to produce noise (i.e., squeaking a small toy or pressing a button on a toy to cause the toy to produce noise such as music or animal sounds.) This modality was coded for when there was no possible way for the other dyad member to have received visual input with the auditory input.

##### Visual

The visual modality included behaviors that somehow incorporated the visual field in getting the other member’s attention. These included waving, gesturing, pointing, making eye contact, holding an object directly in the other member’s visual field, causing a toy to light up (but not produce sound), demonstrating play with toys, offering a toy to the other partner (without using any of the behaviors described in the auditory section), making faces, and changing affect. As no ASL was produced in any of the dyads, it was subsequently excluded from the coding criteria.

##### Tactile

The tactile modality involved using touch, either direct or indirect. Examples included tapping/touching the other person, tickling, hugging, holding, grabbing on to the other person’s clothing, tapping the ground to create vibrations, and touching the other person with a toy (out of their visual field).

##### Auditory-visual

This multimodal classification involved criteria for both the auditory and visual modalities occurring simultaneously. Examples included gesturing while talking, presenting a toy while describing it, reacting to a visual event (e.g., saying “uh oh” when a toy rolls under a table), and demonstrating affect while producing any sort of sound.

##### Auditory-tactile

This multimodal classification included criteria for both the auditory and tactile modalities. This included running a toy over the other partner while making appropriate noises (e.g., running a toy car over the other member’s back while saying “vroom” or making other vehicular noises), holding/grasping hands while signing (e.g., the parent grabs the child’s hands to help them do the motions for “Patty Cake,”), and touching the other person with a toy that made noise.

##### Visual-tactile

This multimodal classification included criteria for visual and tactile modalities. It included behaviors such as taking a toy and making it “hop” up the other person’s arm (without making noise), making eye contact with the other person while also touching them, grabbing the other person’s arm while pointing, and touching the other person with a toy within their visual field while not producing any auditory output.

##### Auditory-visual-tactile

This multimodal classification included simultaneously occurring behaviors encompassed by the criteria for the auditory, visual, and tactile modalities. It included holding a child while pointing and talking to them, making eye contact while singing and touching the other person, and both people playing a clapping game that involves auditory output of some sort while making eye contact.

#### Coding Modality in ELAN

To record modality use in ELAN, the start of the production of a modality was coded in real time (i.e., there was no rule of engagement). However, there was a two second rule of discontinuing the modality, i.e., a participant could pause in production of the modality for up to 2 s and have the subsequent production be part of the same episode. Modality episodes were deemed to be terminated after neither participant engaged in any of the modality criteria behaviors for over 2 s, with the end time of the episode being the end time of the last modality production. Abrupt changes in modality type (e.g., the parent switches from speaking to speaking and pointing) were coded in real time, with no rule of engagement or disengagement.

#### Extracting Data for Analyses

Data were extracted from individual videos using the “View Annotation Statistics” function in ELAN. Total times were extracted for length of time spent in joint attention. In addition, modality times were extracted, after having been coded as a controlled vocabulary in ELAN (and a dependent tier of joint attention). These data were then analyzed as described in Section “Analyses.” Inter-observer reliability (*n* = 3) for these measures was calculated at >90% agreement.

### Analyses

In order to account for differences in the lengths of free play sessions, the metric of proportion of total session length spent in joint attention was computed. To compute this metric, the total amount of time spent in this episode type was extracted from ELAN for each participant. These times were divided by the total session length (excluding uncodeable time) for each participant, i.e., total time spent in adult-initiated, successful bids for joint attention was divided by total session length (see **Table 1** for proportions and lengths of time spent in joint attention for each dyad). With regards to modality, seven modality metrics were computed for both parents and children. This was done by extracting the total amount of time spent in each of the seven modalities, and dividing each in turn by the total amount of time spent in joint attention in the free play session. Mann–Whitney *U* analyses were conducted not only to compare joint attention behavior between the hearing parent-hearing child and hearing parent-deaf child groups, but also to compare modality use by both parents and children in the two dyad types.

## Results

We first compared the overall proportion of time spent in joint attention between parents and children in the two dyad types. The results of a Mann–Whitney *U* analysis indicated that hearing parent-hearing child dyads spent a significantly higher proportion of time in joint attention than hearing parent-deaf child dyads, *U* = 15, *p* < 0.05.

We then evaluated modality use by adults across dyad types during periods of joint attention. Because no instances of tactile-only or auditory-tactile modality combinations were produced by adults in either dyad type, these modalities were excluded from further analysis. First, a comparison of the differences in proportion of time spent in the auditory modality reveals that adult in hearing parent-hearing child dyads spent significantly more time in the auditory modality than adults in hearing parent-deaf child dyads, *U* = 13, *p* < 0.05. Moreover, adults in hearing parent-hearing child dyads spent a greater proportion of time in the visual modality than hearing parent-deaf child dyads, *U* = 14, *p* < 0.05. Thus, hearing parents of hearing children were more likely to use unimodal forms of communication than hearing parents of deaf children (see **Table 2** for a summary of results; see **Table 3** for descriptive statistics).

**Table 2:** Mean ranks of adult modality use.

	Auditory	Visual	Auditory-visual	Visual-tactile	Auditory-visual-tactile
Hearing parent-deaf child	3.3	3	5.5	4.3	6.3
Hearing parent-hearing child	5.8	6	3.5	4.8	2.8

*A higher mean rank indicates that adults in that dyad type spent significantly more time in that particular modality. All results were significant at *p* < 0.05.*

**Table 3:** Descriptive statistics of proportion of time spent by adults in each modality type during joint attention.

	Auditory	Visual	Auditory-visual	Visual-tactile	Auditory-visual-tactile
Hearing parent-deaf child *M* (*SD*)	0.03 (0.06)	0.24 (0.12)	0.46 (0.11)	0.02 (0.04)	0.18 (0.13)
Hearing parent-hearing child *M* (*SD*)	0.11 (0.12)	0.45 (0.14)	0.36 (0.10)	0.01 (0.01)	0.02 (0.04)

What about instances in which two modalities were used during joint attention episodes? A comparison of the proportion of time adults in the two dyad types spent communicating in the auditory-visual modality revealed a significant difference, *U* = 4, *p* < 0.05, such that adults in hearing parent-deaf child dyads spent a greater proportion of time using this combination than hearing parents of hearing children. In contrast, analysis of the visual-tactile modality demonstrated that adults in hearing parent-hearing child dyads spent a significantly greater proportion of time using this combination than adults in hearing parent-deaf child dyads, *U* = 9, *p* < 0.05. Finally, the only case in which adults used three modalities simultaneously involved auditory-visual-tactile communication. In this case, adults in hearing parent-deaf child dyads spent significantly more time in the auditory-visual-tactile modality than adults in hearing parent-hearing child dyads, *U* = 1, *p* < 0.05.

We now turn to analyses of children’s use of different modalities during joint attention. No instances of the tactile modality were observed, so this was excluded from further analysis. Beginning with the auditory modality, results demonstrated—not surprisingly—that children in hearing parent-hearing child dyads (that is, hearing children) spent a significantly greater proportion of time using the auditory modality than children in hearing parent-deaf child dyads (that is, deaf children), *U* = 14, *p* < 0.05. Likewise, deaf children spent a significantly higher proportion of time using the visual modality than hearing children, *U* = 2, *p* < 0.05. These differences in unimodal communication channel make sense and, we would argue, validate our measurement system (see **Table 4** for a summary of results; see **Table 5** for descriptive statistics).

**Table 4:** Mean ranks of child modality use.

	Auditory	Visual	Auditory-visual	Auditory-tactile	Visual-tactile	Auditory-visual-tactile
Hearing parent-deaf child	3	6	3.1	4	3.3	3.9
Hearing parent-hearing child	6	3	5.9	5	5.8	5.1

*A higher mean rank indicates that children in that dyad type spent significantly more time in that particular modality. All results were significant at *p* < 0.05.*

**Table 5:** Descriptive statistics of proportion of time spent by children in each modality type during joint attention.

	Auditory	Visual	Auditory-visual	Auditory-tactile	Visual-tactile	Auditory-visual-tactile
Hearing parent-deaf child *M* (*SD*)	0.00 (0.00)	0.62 (0.12)	0.06 (0.11)	0.00 (0.00)	0.12 (0.14)	0.06 (0.11)
Hearing parent-hearing child *M* (*SD*)	0.07 (0.06)	0.32 (0.17)	0.15 (0.11)	0.003 (0.005)	0.35 (0.35)	0.06 (0.05)

Turning to multimodal comparisons, analyses revealed that hearing children of hearing parents spent a greater proportion of time in the auditory-visual modality than deaf children of hearing parents, *U* = 13.5, *p* < 0.05. A comparison of use of the auditory-tactile combination of modalities revealed that hearing children spent significantly more time using it than deaf children, *U* = 10, *p* < 0.05, as was the case for the visual-tactile combination as well, *U* = 13, *p* < 0.05. Finally, we observed that deaf children spent significantly less time using the auditory-visual-tactile combination than hearing children, *U* = 10.5, *p* < 0.05.

## Discussion

Our results highlight interesting differences in both unimodal and multimodal communication used during episodes of joint attention by parents and children in hearing–hearing and hearing-deaf dyads. Some of the results make sense; others are more surprising and, perhaps, concerning. At the very least, these data demonstrate the variability in accommodation made by parents across different parent–child dyads.

First, we found that hearing parents of hearing children spent a significantly greater proportion of time communicating with their children in both the auditory-only modality and the visual-only modality than hearing parents of deaf children. In other words, hearing parents of hearing children used more unimodal communication during joint attention episodes than hearing parents of deaf children. Of course, the shared use of oral language in hearing parent-hearing child dyads produced a richer body of linguistic interactions overall and, because rich linguistic interactions beget rich attentional interactions, joint attention is no doubt easier for these dyads to establish. The lack of complex, language-based interactions between hearing parents and their deaf children could explain some of the discrepancies in modality use between the two dyad types.

Second, in contrast to previous research showing that hearing parents tend to use the auditory modality most often when trying to engage their deaf children (Koester et al., 1998a), our findings revealed that hearing parents accommodate their deaf children’s hearing status at least somewhat by engaging them via multiple modalities. In particular, adults in hearing parent-deaf child dyads spent a higher proportion of time using the audio-visual modality combination than those in hearing parent-hearing child dyads. However, the reverse pattern was observed for the visual-tactile combination. Why are hearing parents of hearing children spending more time using this combination than hearing parents of deaf children? One possibility is that the hearing children in this study were simply engaged in more physical play, which elicited more tactile interaction with the parent. However, in instances during which three modality combinations were observed, they were produced by hearing parents of deaf children, a finding that is not consistent with such an interpretation. Regardless, the fact that parents in the mismatched dyads were more likely to use multiple modalities during communication than those in matched dyads demonstrates these parents’ effort to accommodate their children’s hearing status.

With regard to children’s use of uni- and multimodal communication, we observed that deaf children spent a greater proportion of time than hearing children using only the visual modality. While this is not surprising given that the visual modality is accessible to a deaf child while the auditory is not, this raises the question of whether children are aware that their parents communicate differently than they do. Another item of note is that hearing children produced the only instances of the auditory-tactile combinations that we observed. When considering the parent and the child data, the overall pattern suggests that hearing parent-hearing child dyads were communicating more in general, an interpretation that is consistent with our finding that hearing parent-hearing child dyads spent a greater proportion of time *in* joint attention relative to hearing parent-deaf child dyads. Of course, the overall amounts of joint attention were small and so we do not wish to make too much of this difference. However, while the present study extends the body of research on this topic by further detailing modality use between the two dyad types, it raises several questions about the nature of communication between hearing parents and their deaf children that merit further investigation. Thus, the preliminary findings of the present study should serve to motivate future research on this issue.

There are several additional factors that necessarily constrain interpretation of our results. First, it is important to note that the sample size is quite small. More observations are needed from more dyads of both types. Another shortcoming is that, although the deaf and hearing children were age-matched, the children are quite varied in age across the dyads. Free play with an 18-month-old is quite different from that with a 36-month-old. Thus, this variability undoubtedly influenced the findings of the present study. Moreover, the hearing parent-deaf child dyad with the oldest child produced the least he amount of joint attention. Why? We can only speculate that this older child found the new toys provided in the study of great interest and willfully chose to focus on the toys rather than the parent. An examination of how parent–child interaction changes over time and relates across time would help clarify some of these questions, as well as facilitate more sophisticated and detailed understanding of the dynamics of age and interaction.

Nonetheless, while the present study has raised more questions than it has answered with regard to modality use in joint attention between parents and their children, it demonstrates that detailed coding of modality use in parent–child communication can provide important insights into how parents accommodate their children’s particular communicative needs, whether they are hearing or deaf. This should motivate additional research of this type. Future studies will be needed to address not only *how* communication is facilitated in joint attention in the two types of dyads, but *what* is going on during these different types of engagement and how it affects the children’s subsequent development.

## Conflict of Interest Statement

The authors declare that the research was conducted in the absence of any commercial or financial relationships that could be construed as a potential conflict of interest.
